# Bedding Application to Feedlot Steers: Influence on Growth Performance, Estimated Maintenance Coefficient, Carcass Characteristics, and Circulating Metabolites in Beef Steers

**DOI:** 10.3390/ani10101766

**Published:** 2020-09-29

**Authors:** Dathan Thomas Smerchek, Zachary Kidd Smith

**Affiliations:** Department of Animal Science, South Dakota State University, Brookings, SD 57007, USA; dathan.smerchek@sdstate.edu

**Keywords:** bedding, feedlot, maintenance coefficient, steers

## Abstract

**Simple Summary:**

A sizeable portion of cattle fed in the United States are fed in the Upper Midwest and Northern Plains where winter temperatures routinely fall below freezing. Using bedding application to improve cattle comfort and performance has been a common practice in livestock production for many years. However, the exact degree to which bedding improves performance is difficult to quantify. As such, two studies were conducted to evaluate the effects of bedding use on growth performance, estimated maintenance energy requirement, carcass characteristics, sera responses in beef steers of differing ages, and phases of feedlot production. Experiment 1 showed that applying wheat straw bedding to yearling crossbred beef steers at a rate of 1.8 kg/steer·d^−1^ increased feed consumption, feed efficiency, and average daily gain. In experiment 2, newly weaned calves bedded with 1.0 kg of wheat straw bedding/steer·d^−1^ tended to consume 4.5% less feed and had a 5.6% improvement in feed efficiency. In both studies, the energy required for maintenance for non-bedded steers was increased. These experiments indicate that bedding application should be considered to improve growth performance and feed efficiency in beef steers by reducing maintenance energy requirements during the feedlot receiving and finishing phases.

**Abstract:**

Two randomized complete block design experiments were conducted to evaluate the effect of bedding use in confined beef steers. Experiment 1 used Simmental × Angus steers (*n* = 240; initial body weight (BW) = 365 ± 22.5 kg). Experiment 2 used newly weaned Charolais × Red Angus steers (*n* = 162; initial BW = 278 ± 13.4 kg). Steers were allotted to one of two treatments: (1) no bedding (NO), or (2) 1.8 kg (Experiment 1) or 1.0 kg (Experiment 2) of wheat straw (as-is basis) bedding/steer·d^−1^ (BED). In Experiment 1, applying bedding improved (*p* ≤ 0.01) dry matter intake (DMI), kg of gain to kg of feed (G:F), and average daily gain (ADG). Bedding reduced (*p* = 0.01) the estimated maintenance coefficient (MQ). Dressing percentage, rib fat, marbling, and yield grade were increased (*p* ≤ 0.03) in NO. Bedding resulted in an increase (*p* = 0.01) in serum insulin-like growth factor I (IGF-I). In Experiment 2, a tendency (*p* = 0.06) for increased DMI for NO was noted. Bedding improved G:F (*p* = 0.01). MQ was elevated (*p* = 0.03) for NO and NO had an increase (*p* = 0.02) in serum concentration of urea-N (SUN). An increase (*p* = 0.01) in serum non-esterified fatty acid was noted for NO. These data indicate that bedding application should be considered to improve growth performance and feed efficiency by reducing maintenance energy requirements in beef steers during the feedlot receiving and finishing phase.

## 1. Introduction

Feeding cattle in the upper Midwest can pose a unique set of environmental challenges. During late fall, winter, and early spring, persistent cold temperatures coupled with snow accumulation, wind, and ice can cause undesirable pen conditions for cattle. These undesirable pen conditions can negatively impact the insulative capacity of cattle hair coat as a result of dampness and mud or manure accumulation. For cattle, the insulative capacity of the hair coat is a contributing factor to their lower critical temperature (LTc) threshold. The LTc for all homeotherms is the temperature below which the organism’s metabolic rate must increase in order to maintain homeothermy [1]. The maintenance requirement of an animal is an estimate of the amount of energy necessary to keep an animal in an equilibrium state [2].Temperatures falling below the lower critical temperature for cattle with a dry, heavy winter coat (~−7.8 °C) will result in a subsequent increase in maintenance requirements and due to this diversion of energy toward maintenance function, a resulting decrease in feed available for gain and productive function is likely to be observed through decreased performance.

Previous work has been done related to the effects of bedding application and housing techniques [3,4,5,6] on beef cattle performance, however, results have been variable with regard to feedlot growth performance and carcass trait responses. Modern performance tracking systems currently used to predict cattle performance rely on two specific requirements of the beef animal, net energy required for maintenance, and net energy for gain [7]. Thus, during winter months, understanding the alteration in maintenance requirement is crucial as it may allow for more accurate tracking and performance prediction.

Little work has been done directly investigating the effects of bedding on receiving phase growth performance in beef steers. The receiving phase is a critical time in beef cattle production that involves a variety of potential stressors. A newly received calf may be exposed to a wide array of stressors including, but not limited to environmental conditions, weaning, transportation, lack of feed and water, and introduction to unfamiliar feed resources [8]. Therefore, mitigating stress by applying bedding may prove valuable when considering newly weaned calf performance in the feedlot. 

The objective of these experiments were to evaluate the effect of bedding use on growth performance (Experiment 1 and 2), carcass characteristics (Experiment 1), estimated maintenance requirement (Experiment 1 and 2), and sera metabolite responses (Experiment 1 and 2) in beef steers of differing ages and during different phases of feedlot production. The hypothesis was that bedding application would increase growth performance and lower estimated maintenance requirement compared to non-bedded steers regardless of stage of production.

## 2. Materials and Methods

### 2.1. Use of Animal Subjects

Animal care and handling procedures used in this study were approved by the South Dakota State University Institutional Animal Care and Use Committee (Approval numbers: 18-096A and 19-054E).

### 2.2. Animal Description and Initial Processing

In Experiment 1, Simmental x Angus crossbred beef steers (*n* = 240; initial BW = 365 ± 22.5 kg) were transported (1.5 h) from a cattle auction facility in eastern South Dakota and received in January of 2019. Steers were allotted to 30 concrete surface pens (7.25 m^2^/steer; 94.5 cm of bunk space/steer; *n* = 8 steers/pen) at the Ruminant Nutrition Center (RNC) in Brookings, SD and provided ad libitum access to long-stem grass hay and water upon arrival. 

Initial processing included an individual body weight measurement (scale readability 0.454 kg), application of a unique identification ear tag, and a rectal temperature measurement along with vaccination for bovine respiratory syncytial virus (BRSV), bovine rhinotracheitis (IBR), bovine viral diarrhea (BVD) Types 1 and 2, parainfluenza-3 (PI3), Mannheimia haemolytica (pasteurella), and clostridium perfringens type A; and administered pour-on moxidectin according to label instructions. Any steer with a rectal temperature of greater than 39.4 °C was administered tulathromycin according to the label’s instructions. On day 36, cattle were implanted with a trenbolone acetate and estradiol benzoate implant and re-vaccinated for clostridium perfringens type A and were poured with an anti-parasitic to control for lice.

In Experiment 2, newly weaned Charolais x Red Angus crossbred beef steers (*n* = 162; initial BW = 278 ± 13.4 kg) were transported (6.0 h) from a sale barn in western South Dakota to the RNC in October of 2019. Upon arrival to the RNC, steers were housed in 18 concrete surface pens (6.45 m^2^/steer; 84.7 cm of bunk space/steer; *n* = 9 steers/pen) with 7.62 m of linear bunk space and provided ad libitum access to long-stem grass hay and water upon arrival. 

The following day (day −1), all steers were individually weighed (readability 0.454 kg), applied a unique identification ear tag, vaccinated for viral respiratory pathogens: IBR, BVD 1 and 2, PI_3_, and BRSV as well as clostridials. The afternoon following initial processing, all steers were allotted to their study pens (*n* = nine steers/pen and nine pens/treatment). The following morning (day 1), all steers were again individually weighed as well as administered pour-on moxidectin according to the label’s directions. On study day 14, all steers were implanted with 200 mg progesterone and 20 mg estradiol benzoate. The initial BW was the average of processing BW (day −1 BW) and day 1 BW. Steers were used to evaluate the effect of bedding application on growth performance and maintenance energy requirements during the feedlot receiving phase. Diets were offered on top of long-stem grass hay (GH) for the first two days of the receiving period. There was no morbidity or mortality noted in Experiment 2. Diets are presented in Table 1 and Table 2 and are composed of actual dry matter (DM) diet composition, actual nutrient concentrations, and tabular energy values [9].

### 2.3. Experimental Design and Treatments

In both experiments, bedding was applied as necessary with the goal of maintaining a dry, bedded area large enough for all steers within the particular bedded pen to lay down. The amount of bedding applied was presented kg per steer per day (as-is basis) of wheat and was calculated as an average based on total kg of bedding applied to the bedded pens throughout the study, divided by days on feed and number of head per pen.

In Experiment 1, pens were assigned to one of two bedding treatments (*n* = 15 pens/treatment): No bedding applied (NO) and 1.8 kg (as-is basis) of wheat straw bedding/steer·d^−1^ (BED). The first nine pen replicates began testing 14 d prior to the last six pen replicates for each treatment due to the timing of acquisition of sufficient cattle to enroll in the experiment. In Experiment 2, pens were assigned to one of two treatments (*n* = nine pens/treatment): No bedding (NO) and 1.0 kg (as-is basis) of wheat straw bedding/steer·d^−1^ (BED). The goal of bedding application, in both experiments, was to maintain a dry, bedded area large enough for all steers to lay down in BED treatment pens at all times during the study. 

### 2.4. Dietary Management 

In both Experiment 1 and 2, fresh feed was manufactured twice daily at 0800 h and 1400 h in a stationary mixer (2.35 m^3^; scale readability 0.454 kg) and bunks were managed according to a slick bunk management approach. Orts were collected, weighed, and dried in a forced air oven at 100 °C for 24 h to determine DM content if carryover feed spoiled or was present on weigh days. If carryover feed was present on weigh days, the residual feed was removed prior to the collection of BW measurements. The DMI of each pen was adjusted to reflect the total DM delivered to each pen after subtracting the quantity of dry orts for each interim period. Actual diet formulation and nutrient composition was determined based upon weekly feed analyses [Crude protein (CP), Official Methods of Analysis (AOAC) (1984); neutral detergent fiber (NDF) and acid detergent fiber (ADF), [10]; ash and DM, [11] and corresponding feed batching records were generated.

In Experiment 1, upon arrival, cattle were stepped up from a 50% to 90% concentrate diet. All pens were on the final high-concentrate diet by day 18. A common diet (Table 1) consisting of dry-rolled corn, dried distillers grains, and oatlage or grass hay was fed that contained 14.2% crude protein, 95.1 Mcal/cwt of net energy for maintenance (NE_M_) and 63.7 Mcal/cwt of net energy for gain (NE_G_). A liquid supplement was provided to add 33 mg/kg of monensin sodium to diet DM along with supplemental vitamins and minerals to meet the National Academies of Sciences, Engineering, and Medicine (NASEM) (2016) requirements. Cattle from BED and NO were on feed 143 and 178 day, respectively, prior to being harvested at a commercial abattoir when the population reached sufficient fat cover to grade United States Department of Agriculture (USDA) Choice. 

Diets in Experiment 2 consisted of corn silage, dried distillers grains plus solubles, grass hay, and a pelleted supplement (Table 2). The diet was fortified with vitamins and minerals to meet nutrient requirements and provided monensin sodium (DM basis) at 27.6 mg/kg [12]. 

### 2.5. Growth Performance Calculations and Carcass Data Collection

In both Experiments 1 and 2, the following equation was used to calculate the estimated maintenance coefficient (MQ) based upon intake, dietary net energy content, and retained energy (RE) required for the observed ADG [13,14].

(1) $$\begin{array}{c} \mathrm{MQ}\left(\mathrm{Mcal}/\mathrm{BW},\;{\mathrm{kg}}^{0.75}\right)=\\ \frac{(\mathrm{DMI},\;\mathrm{kg}-\mathrm{FFG},\;\mathrm{kg}\left[\mathrm{calculated}\;\mathrm{from}\;\mathrm{tabular}\;\mathrm{diet}\;\mathrm{NEg}\;\mathrm{and}\;\mathrm{retained}\;\mathrm{energy}\;\mathrm{required}\;\mathrm{for}\;\mathrm{the}\;\mathrm{observed}\;\mathrm{ADG}\right])\times \mathrm{tabular}\;\mathrm{NEm}}{\mathrm{BW},\;{\mathrm{kg}}^{0.75}} \end{array}$$

In Experiment 1, steers were individually weighed on days −1, 1, 36, 64, 92, and 120 relative to study initiation. Cattle from BED were removed from the experiment where they were then marketed and harvested on d 148 and 134, respectively. The remaining cattle from the group that started 14 d earlier were weighed on d 162 and 183; steers from the group that started 14 d later were weighed on days 148 and 169. Weight gain was based upon initial un-shrunk BW (average of days −1 and 1 BW) and final BW was calculated from hot carcass weight (HCW)/0.625 (a common dressing percentage).

In Experiment 2, all steers were weighed on days −1, 1, 14, 28, 42, and 56. Weight gain was based upon initial un-shrunk on test BW (average of days −1 and 1 BW) and final BW that was pencil shrunk 4% to account for gastrointestinal tract fill. 

In Experiment 1, steers were harvested at a commercial abattoir when the carcass data including ribeye area, hot carcass weight, 12th rib fat, kidney, pelvic, and heart fat percent, and USDA marbling score were collected by the camera grading system at the abattoir. Yield grade was calculated by using the USDA regression equation [15]. Estimated empty body fat (EBF) from carcass traits was calculated according to [16]. Retail yield (RY) as a percentage of HCW was calculated according to [17]. Carcass data were not collected in Experiment 2. Average daily gain was calculated from initial BW subtracted by final BW and divided by the days on feed. Gain to feed ratio was calculated from average daily gain divided by dry matter intake.

### 2.6. Blood Sample Collection

In both experiments, whole blood samples were collected from sentinel steers (*n* = 2 steers/pen) into 10 mL non-additive tubes during the interim weighing process prior to feeding. For Experiment 1, whole blood was collected on days 36, 64, 92, and 120 (relative to study initiation). For Experiment 2, whole blood was collected on days 1, 14, 28, 42, and 56 (relative to study initiation). In both experiments, once collected, whole blood was transported from the RNC to the Ruminant Nutrition Lab, allowed to clot for 24 h at 4 °C, and subsequently centrifuged at 1250× *g* at 4 °C in order to harvest sera.

### 2.7. Serum Hormone and Metabolite Quantification

In Experiment 1, serum concentration of urea-N (SUN) were determined by a method described by Fawcett and Scott [18] using sodium phenate and sodium hypochlorite. The determination of SUN was measured based on the reaction of ammonia with sodium phenate and hypochlorite to yield a blue color to be measured in a spectrophotometer. The SUN assay was performed using serum from each individual steer (*n* = 2 steers/pen) and these values were averaged together prior to statistical analysis. The standard curve constructed for the SUN assay was between 0 and 25.0 mg/dL. Absorbance for reactions of standards and samples were read at 625 nm. Samples were considered for re-runs if the coefficient of variation (CV) was greater than 10% among triplicate determinations. Intra- and inter-assay CV were 6.3% and 10.9%, respectively. 

In Experiment 1, serum concentrations of insulin-like growth factor I (IGF-I) were determined in duplicate via radioimmunoassay procedure [19,20]. Insulin-like growth factor binding proteins (IGFBP) in sera were extracted using a 1:17 ratio of sample to acidified ethanol (12.5% 2 N HCl: 87.5% absolute ethanol) [21]. Extracted samples were centrifuged (12,000× *g* at 4 °C) to separate IGFBP. A portion of the resulting supernatant was removed and neutralized with 0.855 M Tris base, incubated for an additional 4 h at 4 °C, and then centrifuged at 12,000× *g* at 4 °C to remove any additional IGFBP. When samples of this extract, equivalent to the original serum sample, were subjected to western ligand blot analysis and subsequent phosphoimagery, no detected binding of I-IGF-I to IGFBP was observed. Inhibition curves of the neutralized extracted serum ranging from 12.5 to 50 µL were parallel to the standard curve. Recombinant human IGF-I (GF-050; Austral Biological, San Ramon, CA, USA) was used as the standard and radioiodinated antigen. Antisera AFP 4892898 (National Hormone and Peptide Program, National Institutes of Diabetes, Digestive and Kidney Diseases, Bethesda, MD, USA) was used at a dilution of 1:62,500. Sensitivity of the assay was 14.7 pg/tube. No samples were considered for re-runs and the assay was completed in a single run. The intra-assay CV was 7.7%.

In Experiment 2, the quantification of circulating SUN concentration was determined on a microplate spectrophotometer in triplicate 5 µL determinations using diacetylmonoxime via a commercially available kit (STANBIO Urea Nitrogen-0580; STANBIO Laboratory, Boerne, TX, USA). The SUN assay was performed using serum from each individual steer (*n* = 2 steers/pen) and these values were averaged together prior to statistical analysis. The standard curve constructed for the SUN assay was between 0 and 25.0 mg/dL. Absorbance for reactions of standards and samples were read at 520 nm. Samples were considered for re-runs if the coefficient of variation among the absorbance values for triplicate determinations was greater than 5%. For the SUN analysis in Experiment 2, the intra-assay CV was 6.6% and the inter-assay CV was 10.4%. 

In Experiment 2, quantification of serum concentration of non-esterified fatty acids (NEFA) was determined using triplicate 5 µL determinations via colorimetric assay using a commercially available kit that involved acyl-CoA stynthetase, acyl-CoA oxidase, and perioxidase in 96-well microtiter plates (NEFA-HR; Wako Diagnostics, Richmond, VA, USA). The NEFA assay was performed using sera from each individual steer (*n* = 2 steers/ pen) and these values were averaged together prior to statistical analysis. The standard curve constructed for the NEFA assay was between 0 and 1.0 mEq/L. Samples were considered for re-runs if the coefficient of variation among the absorbance values for triplicate determinations was greater than 5%. For the NEFA analysis, the intra-assay and inter-assay CV were 3.6% and 3.7%, respectively. 

### 2.8. Management of Pulls and Removals

All steers that were pulled from their home pen for health evaluation were then monitored in individual hospital pens prior to being returned to their home pens. When a steer was moved to a hospital pen, the appropriate amount of feed from the home pen was removed and transferred to the hospital pen. If the steer in the hospital returned to their home pen, this feed remained credited to the home pen. If the steer did not return to their home pen, all feed that was delivered to the hospital pen was deducted from the feed intake record for that particular pen back to the date the steer was hospitalized. Eight steers were removed during the course of the experiment for reasons determined to be health anomalies not related to treatment. Six steers from NO were removed due to pneumonia (1), bloat (1), identified as a bull (1), and musculoskeletal issues (3). Two steers from BED were removed due to being identified as bulls. 

### 2.9. Statistical Analysis

Data were analyzed using the GLIMMIX procedure of SAS 9.4 (SAS Inst. Inc., Cary, NC, USA). Experiments 1 and 2 were both randomized complete block designs. Fixed effects included in the model for Experiment 1 were bedding treatment and block (pen location). Fixed effects in Experiment 2 included in the model were bedding treatment and block (pen location). The pen served as an experimental unit for all analyses in both studies; an α of less than 0.05 determined significance and an α between 0.051 and 0.10 was considered a tendency.

Serum metabolite data were analyzed according to a randomized complete block design appropriate for repeated measures using the MIXED procedure of SAS 9.4 (SAS Inst. Inc.). The model included the fixed effects of bedding, day, and their interaction. Day was included as the repeated variable and pen served as the experimental unit. The covariance structure with the lowest Akaike information criterion was used. All results were reported as least squares means. An α of 0.05 determined significance and an α of 0.06 to 0.10 was considered a tendency.

## 3. Results

### 3.1. Weather

**Figure 1:**
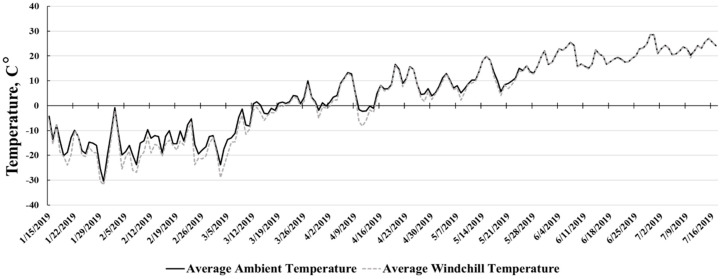
Experiment 1: Cumulative average ambient temperature (C°) and average wind chill temperature (C°) during the study (15 January 2019 to 17 July 2019).

**Figure 2:**
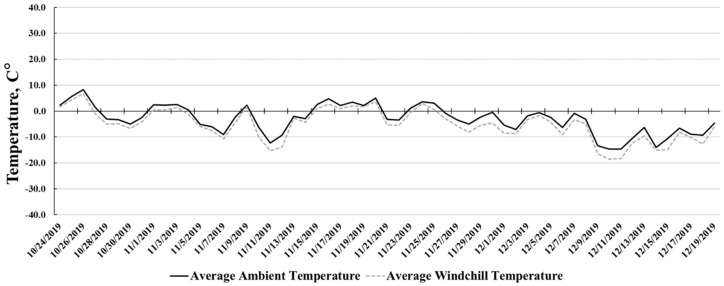
Experiment 2: Cumulative average ambient temperature (°C) and average wind chill temperature (°C) during the study (24 October 2019 to 19 December 2019).

Experiment 1 was conducted from 15 January to 17 July 2019. Daily ambient temperature (Figure 1) averaged 4.4 ± 14.6 °C with an average wind chill of 2.9 ± 15.8 °C during the course of the study. Experiment 2 was conducted from October to December of 2019. Daily ambient temperature (Figure 2) averaged −3.0 ± 5.5 °C and wind chill averaged −5.1 ± 6.1 °C during the 56 days receiving study.

### 3.2. Growth Performance—Experiment 1

#### 3.2.1. Initial—Day 1 to Day 36

Growth performance and carcass data from Experiment 1 are located in Table 3. During the receiving phase of Experiment 1 (d 1 to 36), weather was more severe than the remainder of the study. Initial BW did not differ (*p* = 0.95) between NO and BED. Dry matter intake was not affected (*p* = 0.57) by bedding treatment and d 36 BW was greater for BED (*p* = 0.01; 419 vs. 402 ± 1.1 kg) compared to NO. A 48.0% increase (*p* = 0.01) in receiving phase ADG and a 49.2% increase in receiving phase G:F (*p* = 0.01) was observed in BED compared to NO. An increase (*p* = 0.01) in MQ was noted for NO (0.146 vs. 0.104 ± 0.0032 Mcal/ BW^0.75^, kg) relative to BED.

#### 3.2.2. Cumulative

In Experiment 1, final BW tended to differ (*p* = 0.07) between NO and BED. Dry matter intake was increased (*p* = 0.01) by 5.8% in BED compared to NO. Cumulative ADG (*p* = 0.01) and G:F were improved (*p* = 0.01) in BED by 21.0% and 15.0%, respectively. The cumulative estimated maintenance coefficient was elevated (*p* = 0.01; 0.109 vs. 0.098 ± 0.010 Mcal/BW^0.75^, kg) for NO compared to BED steers. 

#### 3.2.3. Carcass Characteristics—Experiment 1

Hot carcass weight tended to differ (*p* = 0.07) between NO and BED. Cattle from NO required an additional 35 days to achieve similar final live-basis BW. Rib eye area (*p* = 0.69) did not differ between NO and BED. Dressing percentage, rib fat, marbling, and yield grade were increased (*p* ≤ 0.03) in NO steers compared to BED.

#### 3.2.4. Serum Hormones and Metabolites—Experiment 1

**Figure 3:**
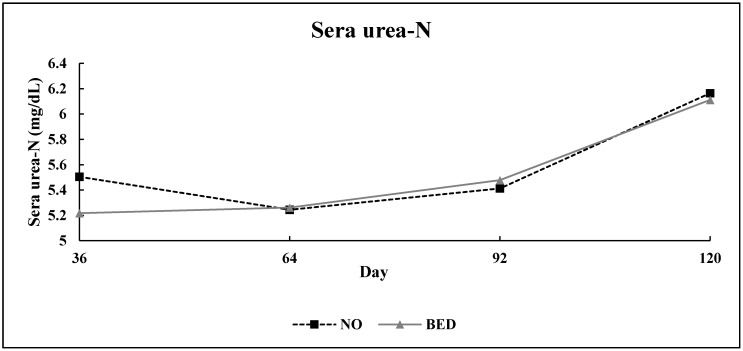
Experiment 1: Effect of bedding treatment on serum concentration of urea-N (SUN) in finishing steers (*n* = 15 pens/treatment; pooled bed × day SEM (Standard error of the mean) = 0.23). Treatments were: No bedding applied (NO) and 1.8 kg (as-is basis) of wheat straw bedding/steer·d^−1^ (BED). Blood was collected and harvested as sera on days 36, 64, 92, and 120.

No bed × day interaction (*p* = 0.66) was detected for SUN concentration in Experiment 1 (Figure 3). The main effect of bedding treatment did not cause a significant response (*p* = 0.75) in SUN between treatments, however, SUN did differ over time (*p* = 0.01).

**Figure 4:**
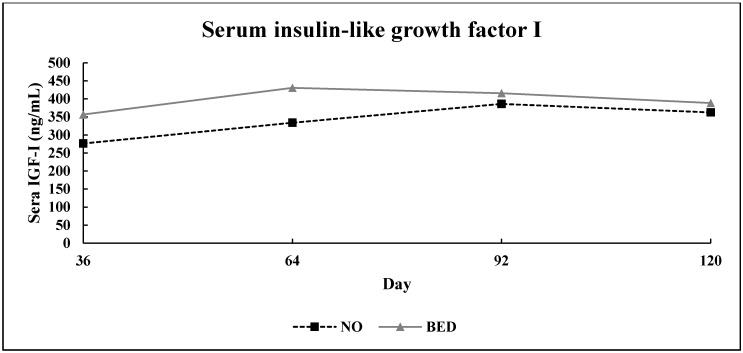
Experiment 1: Effect of bedding treatment on serum concentration of insulin-like growth factor I (IGF-I) in finishing steers (*n* = 15 pens/treatment; pooled bed × day SEM (Standard error of the mean) = 25.71). Treatments were: No bedding applied (NO) and 1.8 kg (as-is basis) of wheat straw bedding/steer·d^−1^ (BED). Blood was collected and harvested as sera on days 36, 64, 92, and 120.

No bed × day interaction (*p* = 0.24) was detected for serum concentration of IGF-I (Figure 4). Bedding treatment resulted in a 17% increase (*p* = 0.01) in sera IGF-I. Circulating sera IGF-I also differed over time (*p* = 0.01).

### 3.3. Growth Performance—Experiment 2 

#### 3.3.1. Cumulative 

Growth performance responses for Experiment 2 are located in Table 4. Initial BW did not differ (*p* = 0.69) between treatments at study initiation. Bedding application did not influence (*p* ≥ 0.67) final BW or ADG. Dry matter intake tended to increase (*p* = 0.06) in NO steers relative to the BED. Gain to feed was increased (*p* = 0.01) by 5.6% for cattle in bedded pens relative to NO. Estimated MQ was elevated (*p* = 0.03; 0.052 vs. 0.044 ± 0.0022 Mcal/BW^0.75^, kg) for NO steers compared to BED steers.

#### 3.3.2. Serum Metabolites—Experiment 2 

**Figure 5:**
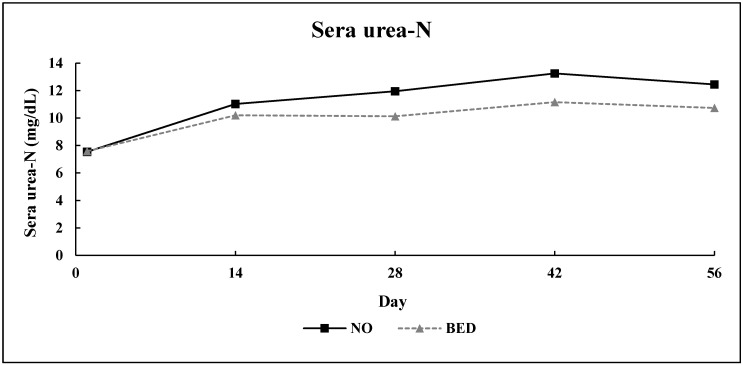
Experiment 2: Effect of bedding treatment on serum concentration of urea-N (SUN) in finishing steers (*n* = 9 pens/treatment; pooled bed × day SEM (Standard error of the mean) = 0.82). Treatments were: (1) no bedding (NO) or (2) 1.0 kg (as-is basis) of wheat straw bedding/steer·d^−1^ (BED). Blood was collected and harvested as sera on days 1, 14, 28, 42, and 56.

No bed × day interaction (*p* = 0.67) was detected for SUN concentration in Experiment 2 (Figure 5). The main effect of bedding treatment resulted in a 13% increase (*p* = 0.02) in SUN for NO compared to BED. Additionally, SUN differed over time (*p* = 0.01).

**Figure 6:**
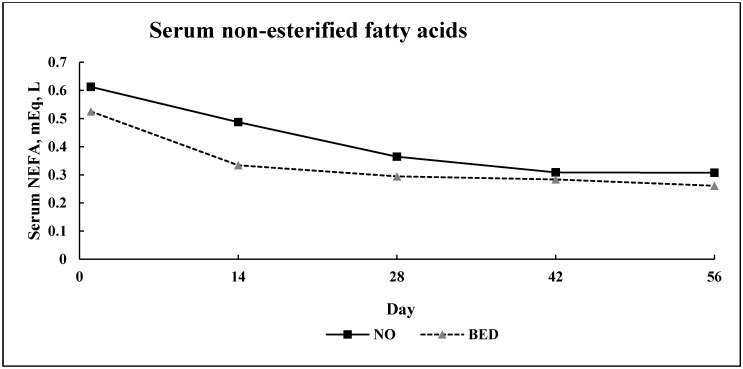
Experiment 2: Serum concentration of non-esterified fatty acids (NEFA) in finishing steers (*n* = 9 pens/treatment; pooled bed × day SEM (Standard error of the mean) = 0.038). Treatments were: (1) no bedding (NO) or (2) 1.0 kg (as-is basis) of wheat straw bedding/steer· d^−1^ (BED). Blood was collected and harvested as sera on days 1, 14, 28, 42, and 56.

No bed × day interaction (*p* = 0.52) was detected for serum NEFA concentration (Figure 6). Bedding treatment resulted in a 22% increase (*p* = 0.01) in serum concentration of NEFA in NO compared to BED steers. Serum concentration of NEFA also differed over time (*p* = 0.01).

## 4. Discussion

### 4.1. Experiment 1

#### 4.1.1. Growth Performance Day 1 to Day 36

Little work has been done to directly investigate the effects of bedding application on feedlot growth performance, and specifically, the resulting alterations in energetic demand. Interim performance data from the initial 36 day receiving period of Experiment 1 were included to better illustrate the effects of the severe environmental conditions (Figure 1) on receiving phase growth performance. This is of importance because earlier work [22,23] determined that growth performance improvements observed during the receiving phase can often be maintained during subsequent feeding periods. 

At the conclusion of the initial 36 d receiving period, a 4.0% increase in d 36 BW was observed for BED steers, which amounted to approximately 17 kg of additional BW gain during the initial 36 day period. A 48.0% increase in ADG was noted for the BED treatment during the 36 day receiving period relative to the NO steers. Interim performance data for BW and ADG, as a result of bedding application, have been reported in previous work, but results have varied. In a study that investigated the effects of bedding level on cattle performance, Anderson, Wiederholt, and Schoonmaker [5] reported that during winter months, both modest and generous amounts of bedding applied during the start of the feeding period resulted in an approximately 20% increase in ADG. Alternatively, in a study that investigated both bedding and shelter effects, Mader and Colgan [6] reported that bedding application during winter months did not result in any appreciable response in BW or ADG at the conclusion of the initial 36 day period. The variation in effects on performance due to bedding application can likely be explained by the large number of external factors that play a pivotal role in the occurrence and magnitude of performance results. These factors may include geographical location, temperature, wind, precipitation, time of year, pen size, stocking density, hair coat condition of animals included in the study, age of animal, and many other possible factors. Performance results from the present study, specifically the initial feedlot receiving period of days 1 to 36, are likely of greater magnitude due to the persistent exposure of the cattle to abnormally low ambient temperatures and severe wind chill. 

Bedding application had no effect on DMI in the initial 36 days period as both treatments consumed similar amounts of dry matter. Intakes were controlled by the feedlot manager as cattle were being stepped up to the high concentrate finishing diet. With no difference in DMI between treatments and significant responses in both d 36 BW and ADG favoring the BED treatment during the initial 36 days period, a 49.2% improvement in G:F ratio was observed in BED steers. It has been well documented that cold temperatures cause an increase in metabolic demand of beef cattle [1,3,6,24], and so if cattle are not able to compensate by consuming more DMI, a resulting decrease in feed efficiency will likely be observed.

It was during the initial 36 days period that the magnitude of difference in MQ was largest between treatment groups. As a response to winter weather conditions such as sustained cold temperatures, snow accumulation, and wind, beef cattle are well known to have increased maintenance requirements in order to maintain homeothermy [1,25]. This principle has been demonstrated in a number of previous studies dealing with bedding application and cold stress [5,6,26]. During the initial 36 d period of Experiment 1, relative to the BED treatment, NO had a MQ that was elevated 40.4%. It should be noted that the severe environmental conditions during the initial 36 days period experienced by all cattle on test, regardless of treatment, caused an increase in their maintenance energy requirements relative to the standard NEm requirement value for beef cattle of 0.077 Mcal/BW^0.75^ [7]. The increases in MQ for NO and BED relative to the standard value of 0.077 Mcal/BW^0.75^ were 90% and 35%, respectively. In a case study by Wagner, Grubb, and Engle [24], data indicated that NEm required by cattle during and in the aftermath of a major winter weather event may be 2.5 times higher than NEm required under standard thermoneutral conditions. These results indicate that, regardless of bedding application and pen surface condition, severe weather events can cause alterations in the energetic demand of beef cattle and thus an increase in feed required for maintenance.

#### 4.1.2. Cumulative Growth Performance

In Experiment 1, there was a tendency for final BW to differ between NO and BED, however it should be noted that steers from NO remained on feed for an additional 35 d to achieve a similar compositional endpoint as BED steers. It is probable that, had cattle been marketed at equal days on feed, final BW would have favored the BED treatment. In some previous work, during winter and spring months, final BW was increased in bedded treatments compared to non-bedded controls when cattle were marketed at equal days on feed [3,5]. Steer ADG was improved in BED by 21.0% compared to the NO control steers. Mader [27], along with a number of other studies [3,4,5], reported increases in ADG as a result of bedding application. However, other work previously reported did not observe increases in ADG as a result of bedding application [6]. As it relates to feedlot cattle, cold temperatures are well known to increase energy required for maintenance, increase rate of passage, and stimulate appetite in cattle as a response to the increased metabolic demands [1]. In the present study, cattle from BED treatment consumed 5.8% more DMI than cattle from NO. Previous work conducted regarding the effects of bedding application on feedlot growth performance during winter months did not report any differences in DMI as a result of bedding application [3,4,5,6]. The difference observed in DMI that favored the BED treatment could be a lasting effect resulting from increased growth performance captured during the initial 36 d period of the study. As stated previously, growth performance improvements observed during the receiving phase can often be maintained during subsequent feeding periods [22,23]. Overall G:F was improved in BED cattle by 15.0% compared to NO. A common physiological reaction of ruminants, when exposed to cold stress, has been shown to be increased reticulorumen motility and rate of passage of digesta [28]. This physiological response may, in part, account for the observed disparity in feed efficiency. The improvement in feed efficiency for the BED treatment observed in the present study as a result of bedding treatment is consistent with previous work [3,5,6], although the degree to which feed efficiency improved in these previous studies varied, likely because of geographical location and weather conditions.

Estimated maintenance coefficient was elevated 11.2% for NO compared to BED, which was similar to previous findings [5,6]. The estimated maintenance coefficient for steers in BED pens compared to NO can likely be explained as a function of the performance results previously reported and discussed for Experiment 1 where BED cattle required fewer days on feed (DOF), consumed more dry matter, and had improved ADG and G:F. Bedding application appears to have decreased the proportion of metabolizable energy (ME) intake partitioned to maintenance functions, when compared to NO, which allowed a greater proportion of ME intake to be used for productive function and stored as retained energy (RE) rather than heat production to maintain homeothermy. Both NO and BED treatments had increased MQ relative to the 0.077 Mcal/BW^0.75^ value from Lofgreen and Garrett [7].

The effects of bedding on beef cattle feedlot performance are inherently linked to the environmental conditions experienced by the cattle being evaluated. The unavoidable variation in pen condition, geographical location, and weather conditions pose considerable challenges when attempting to compare performance results from previous work. Additionally, potential long-term effects on growth performance as a result of exposure to extreme winter temperatures, like those environmental conditions experienced by steers during the first 36 days of Experiment 1, in a non-bedded versus bedded pen environment, requires further investigation.

#### 4.1.3. Carcass Characteristics—Experiment 1

There was a tendency for NO steers to have heavier HCW compared to and the BED steers. Anderson, Wiederholt, and Schoonmaker [5], in a study evaluating effects of bedding level on feedlot cattle performance, reported that “generous” bedding level improved HCW in bedded pens for cattle fed for equal days. However, in previous work, other authors [4,6] reported no effect on HCW for beef cattle fed for equal days. In the present study, had cattle been harvested at an equal number of days on feed, it is likely that a response in HCW favoring BED cattle would have been noted given cattle from NO required an additional 35 d to achieve final live BW similar to that of the BED treatment. Conversely, perhaps an explanation to oppose this idea, is that during this experiment, an inadvertent increase in frame size occurred in NO treatment due to a decreased amount of feed available for gain as a result of the increased calculated maintenance coefficient during the early periods of this experiment. Rib eye area did not differ between NO and BED. This result is inconsistent with Anderson, Wiederholt, and Schoonmaker [5], who reported a significant increase in REA for bedded steers compared to non-bedded controls fed for equal days. Limited additional data are available reporting the effect of bedding on REA in beef steers. Dressing percentage was increased for the NO treatment, differences in manure tag load, frame size, gut fill, and days on feed may explain this response. The dressing percentage response favoring the NO treatment, in the present study, was inconsistent with previous work where bedded treatments had improved dressing percentages compared to non-bedded control treatments [4,5]. Mader and Colgan [6] reported that bedding did not cause a significant response in dressing percentage in either of their two trials. 

In the present study, rib fat was increased for NO steers compared to BED. This is inconsistent with findings from Anderson, Wiederholt, and Schoonmaker [5], who reported no difference in rib fat as a result of bedding application. Additional work reporting the effect of bedding on rib fat in beef steers is currently limited. Marbling score was also improved in NO steers compared to BED. Mader and Colgan [6] reported no difference in marbling score as a result of bedding application in both bedding trials. Anderson, Wiederholt, and Schoonmaker [5] reported a significant response in marbling score favoring bedded cattle. In another experiment, Anderson, Wiederholt, and Schoonmaker [5] did not observe an effect on marbling score. In the present study, a response was noted where NO steers had increased calculated yield grade compared to BED steers. This is likely a function of increased rib fat and estimated empty body fat [16]. Anderson, Wiederholt, and Schoonmaker [5] reported increased calculated yield grade for bedded cattle compared to non-bedded controls. Other workers reported no effect of bedding on calculated yield grade [4,6].

#### 4.1.4. Serum Hormones and Metabolites—Experiment 1

Serum concentration of urea-N was not affected by bedding treatment in Experiment 1. However, SUN did differ over time. The SUN concentration was at its lowest point from d 36 and 64 and then increased on d 92 and 120. The observed decrease from d 36 and 64 for serum concentration of urea-N, indicative of anabolism, may have been caused by the additive effects of increased intakes, implantation on d 36, and perhaps improved weather conditions as the study progressed. 

Bedding treatment, in the experiment, resulted in a 17% increase in the serum concentration IGF-I. Insulin-like growth factor I is a somatotropin-dependent anabolic peptide that stimulates proliferation and differentiation of many cell types including muscle [29]. Therefore, changes in serum concentration of IGF-I were likely a factor that improved growth rate in BED steers and caused them to reach harvest 35 d sooner than NO steers. Serum concentration of IGF-I differed over time, perhaps a function of improving weather conditions where bedding treatment became less important. 

### 4.2. Experiment 2 

#### 4.2.1. Growth Performance

Previous receiving phase growth performance data investigating effects of bedding application are limited. 

In the present study, bedding application did not influence final BW. Previous studies have reported interim data that can be used to compare receiving phase performance results seen in the present study. In two bedding related research trials using cattle with initial BW of 329 kg and 296 kg, respectively, Anderson, Wiederholt, and Schoonmaker [5] reported no difference in d 56 BW. Mader and Colgan [6] also conducted a pair of trials related to effect of bedding on feedlot performance. Body weights were reported for d 35 and d 34 for trials 1 and 2, respectively. In trial 1, where the initial BW of cattle was 373 kg, a significant response in BW was not reported. In trial two, cattle (initial BW = 400 kg) from the bedded treatment had a significantly increased d 34 BW. No improved response was observed for ADG) in the present study. With equal initial BW and no change in final BW, a response in ADG was not expected. This response is inconsistent with some previous work [4,5,6] where enhanced responses for ADG were observed during the early periods of the respective trial. However, Mader and Colgan [6] did report an increased response in ADG favoring the bedded treatment. There was a tendency for NO steers to have a 4.5% increase in dry matter intake (DMI) compared to BED steers in Experiment 2. This agrees with results reported from trial 2 by Anderson, Wiederholt, and Schoonmaker [5], where non-bedded cattle consumed a greater amount of DMI. However, other work has reported no effect on DMI [4,6]. The decrease in DMI for BED steers compared to NO may, in part, be attributable to the consumption of the bedding material. However, it may also be due to decreased maintenance requirements for the BED steers as a result of bedding application. 

Overall G:F was increased 5.6% for BED steers relative to NO steers in Experiment 2. Steers from the BED treatment tended to consume less DMI throughout the 56 d receiving period, but had equal final BW and ADG, subsequently, allowing for greater G:F. Anderson, Wiederholt, and Schoonmaker [5] did not report a difference in d 56 G:F in trial 1, however, G:F was significantly increased for the bedded treatment in trial 2. Mader and Colgan [6] reported no improvements in G:F during the initial periods of trial 1 and 2. The MQ in Experiment 2 was elevated by 18% for NO compared to BED. Daily ambient temperature averaged −3.0 ± 5.5 °C and windchill averaged −5.1 ± 6.1 °C during the 56 d receiving study. Temperatures during Experiment 2 were not as severe as the initial 36 d period in Experiment 1. However, an 18% cumulative increase in MQ was still noted for NO steers compared to BED. Cold temperatures are well known to increase the maintenance requirement of beef cattle [1,25], and this has been demonstrated in a number of previous studies [5,6,26]. In the present study, steers from NO had increased maintenance requirements relative to BED. Bedding application likely lessened the increase in maintenance energy costs in BED steers by providing improved comfort and insulative protection to conserve body heat as well as mitigating some of the stress commonly experienced by cattle during the receiving phase.

#### 4.2.2. Serum Metabolites 

A 13% decrease in SUN concentration was noted for BED steers compared to NO. Concentration of SUN is often used as an indicator of metabolic status in beef cattle with regard to anabolism or catabolism of lean tissue. The observed decrease in SUN may be attributable to the bedding application which, perhaps, aided in stress mitigation via improved comfort and lowered the calculated maintenance coefficient for BED steers, thus, more energy was available for anabolism of lean tissue. Additionally, SUN differed over time. This is perhaps a result of lower temperatures later in the receiving period. Elevated serum NEFA is an indicator of adipose tissue catabolism. Not applying bedding during the 56 d receiving study resulted in a 22% increase in serum concentration of NEFA for NO steers compared to BED. The increase in serum concentration of NEFA for NO steers is likely to be a further indication that BED cattle, due to their lower calculated maintenance coefficient, spent less time in a negative energy balance, and thus did not catabolize adipose tissue in the same manner as the NO steers. Serum concentration of NEFA also decreased over time for both treatments. This decrease over time is expected as even healthy newly received calves, during the first week post-arrival, consume approximately 1.6% of BW. In addition to relatively low intakes, newly received calves encounter a large variety of stressors during this period such as weaning, adverse environmental conditions, transportation, lack of feed and water, and introduction to unfamiliar feed resources [8]. Therefore, these stressors are a likely explanation for serum concentration of NEFA initially being elevated for both treatments and subsequently decreasing throughout the 56 d receiving study.

## 5. Conclusions

In Experiment 1, applying wheat straw bedding to yearling crossbred beef steers at a rate of 1.8 kg/steer·d^−1^ increased DMI, G:F, and ADG. Bedding cattle also reduced the estimated MQ during the entirety of the trial by 11.2%. In Experiment 2, newly weaned receiving calves bedded with 1.0 kg of wheat straw bedding/steer·d^−1^ tended to consume 4.5% less dry matter, and had a 5.6% improvement in G:F. Additionally, MQ was elevated 18% in the non-bedded treatment. These data indicate that, depending on geographical location, cost of bedding, and weather conditions, bedding application should be considered to improve growth performance and feed efficiency in beef steers by reducing maintenance energy requirements during the feedlot receiving and finishing phases.

## Figures and Tables

**Table 1:** Experiment 1: Diet composition (DM basis) ^a^.

	Finisher 1	Finisher 2 ^e^
Item		
Dry-rolled corn, %	69.70	70.33
Dried distillers grains, %	17.00	16.85
Oatlage, %	8.37	-
Grass Hay, %	-	7.89
Liquid supplement ^b^, %	4.93	4.93
Nutrient Composition ^f^		
Dry Matter, %	77.50	85.26
Crude Protein, %	14.20	12.88
Neutral detergent fiber, %	16.60	17.76
Acid detergent fiber, %	6.84	7.14
Ash, %	5.25	5.30
NEm ^c^, Mcal/kg	2.10	2.10
NEg ^d^, Mcal/kg	1.40	1.40

^a^ All values except dry matter on a dry matter (DM) basis; ^b^ Liquid supplement: formulated to add 30 g/t of monensin to diet DM and vitamins and minerals to meet or exceed the National Academies of Sciences, Engineering, and Medicine (NASEM) (2016) requirements; ^c^ Net energy for maintenance; ^d^ Net energy for gain; ^e^ Diet fed for final 12-day of the study when oatlage Fsupply was depleted; ^f^ Tabular NE from [9] and actual nutrient compositions from weekly assay of individual dietary ingredients and feed batching records.

**Table 2:** Experiment 2: Diet composition (DM basis) ^a^.

Item	
Corn Silage ^b^	63.69
Dried distillers grains plus solubles	20.31
Grass Hay	10.00
Pelleted Supplement ^c^	6.00
*Soybean Meal*	*(3.777)*
*Soybean Hulls*	*(0.353)*
*Trace Mineralized Salt*	*(0.30)*
*Calcium Carbonate*	*(1.11)*
*Premix ^d^*	*(0.072)*
Nutrient Composition ^e^	
Dry Matter, %	41.99
Crude Protein, %	13.09
Neutral detergent fiber (NDF), %	40.00
Acid detergent fiber (ADF), %	28.17
Ash, %	6.29
Net energy for maintenance (NE_M_), Mcal/kg	1.74
net energy for gain (NE_G_), Mcal/kg	1.12

^a^ All values except dry matter on a dry matter (DM) basis; ^b^ Corn silage (*n* = 9 samples) contained (DM basis, except for dry matter): 31.50% dry matter, 6.18% crude protein, 39.50% NDF, 30.22% ADF, and 4.58% ash; ^c^ Inclusion to total diet DM included in parentheses; ^d^ Vitamin premix contained (in each 907-kg of supplement): 7204 g of soybean meal (SBM), 1972 g of Rumensin-90 (Elanco, Indianapolis, IN, USA), 48 g of vitamin A (650,000 IU/g), 750 g of vitamin E (500 IU/g), 721 g of IntelliBond Zn (Micronutrients, Indianapolis, IN, USA), and 195 g IntelliBond Cu (Micronutrients) for 0% GH; 7123 g of SBM, 2022 g of Rumensin-90 (Elanco), 49 g of vitamin A (650,000 IU/g), 769 g of vitamin E (500 IU/g), 726 g of IntelliBond Zn (Micronutrients), and 201 g IntelliBond Cu (Micronutrients) for 10% GH; 7226 g of SBM, 1980 g of Rumensin-90 (Elanco), 48 g of vitamin A (650,000 IU/g), 753 g of vitamin E (500 IU/g), 699 g of IntelliBond Zn (Micronutrients), and 184 g IntelliBond Cu (Micronutrients) for 20% GH; ^e^ Tabular NE from [9] and actual nutrient compositions from weekly assay of individual dietary ingredients and feed batching records.

**Table 3:** Experiment 1: Effect of bedding on cattle growth performance and carcass characteristics ^a^.

Item	Bedding Treatment		SEM	*p*-Values
	NO	BED		
Pens, *n*	15	15	-	-
Initial Growth Performance (d 1—36)				
Initial body weight (BW), kg	365	365	0.4	0.95
d 36 BW	402	419	1.5	0.01
Average daily gain (ADG), kg/day	1.02	1.51	0.044	0.01
Dry matter intake (DMI), kg/day	8.19	8.22	0.047	0.57
ADG/DMI, kg/kg	0.124	0.185	0.0047	0.01
Maintenance Coefficient, Mcal/W^0.75^	0.146	0.104	0.003	0.01
Cumulative Growth Performance (d 1—harvest)				
Days on Feed	178	143	-	-
Final Shrunk BW, kg ^b^	575	569	2.0	0.07
Average daily gain (ADG), kg/day	1.18	1.43	0.019	0.01
Dry matter intake (DMI), kg/day	9.30	9.84	0.124	0.01
ADG/DMI, kg/kg	0.127	0.146	0.002	0.01
Maintenance Coefficient, Mcal/W^0.75^	0.109	0.098	0.010	0.01
Carcass Characteristics				
Dressing percentage, %^c^	63.29	62.30	0.140	0.01
Hot carcass weight (HCW), kg	359	356	1.3	0.07
Ribeye area, cm^2^	83.16	82.71	0.76	0.69
Rib fat, cm	1.20	1.09	0.02	0.01
Marbling ^d^	475	437	6.6	0.01
Estimated empty body fat, % ^e^	28.95	28.29	0.140	0.01
Calculated yield grade	2.95	2.81	0.045	0.03
Retail yield, % ^f^	50.53	50.92	0.100	0.01

^a^ Treatments: No bedding applied (NO), 1.8 kg (as-is basis) of wheat straw/steer·d-1 (BED); ^b^ Calculated from hot carcass weight (HCW)/0.625; ^c^ HCW/final BW shrunk 4 %; ^d^ 400 = Small00 (United States Department of Agriculture (USDA) Low Choice); ^e^ According to Guiroy et al. (2002); ^f^ As a percentage of HCW according to Murphey et al. (1960). SEM: Standard error of the mean.

**Table 4:** Experiment 2: Effect of bedding on cattle growth performance ^a^.

Item	Bedding Treatment		SEM	*p*-Values
	NO	BED		
Pens, *n*	9	9	-	-
Days on feed	56	56	-	-
Growth Performance (day 1–56)				
Initial body weight (BW), kg	278	278	0.22	0.69
Final Shrunk BW ^b^	353	355	2.2	0.70
Average daily gain (ADG), kg/day	1.36	1.38	0.04	0.67
Dry matter intake (DMI), kg/day	6.9	6.6	0.09	0.06
ADG/DMI, kg/kg	0.198	0.209	0.005	0.03
Maintenance Coefficient, Mcal/W^0.75^	0.052	0.044	0.002	0.03

^a^ Treatments: No bedding applied (NO) and 1.0 kg (as-is basis) of wheat straw bedding/steer·d-1 (BED); ^b^ Final body weight (BW) was BW from day 56 that was pencil shrunk 4% to account for gastrointestinal tract fill. SEM: Standard error of the mean.
